# Testing of Piezo-Actuated Glass Micro-Membranes by Optical Low-Coherence Reflectometry

**DOI:** 10.3390/s17030462

**Published:** 2017-02-25

**Authors:** Sabina Merlo, Paolo Poma, Eleonora Crisà, Dino Faralli, Marco Soldo

**Affiliations:** 1 Dipartimento di Ingegneria Industriale e dell’Informazione, Università degli Studi di Pavia, 27100 Pavia, Italy; itsamonster87@gmail.com (P.P.); eleonora.crisa01@universitadipavia.it (E.C.); 2 STMicroelectronics, 20864 Agrate Brianza (MB), Italy; dino.faralli@st.com; 3 STMicroelectronics, 20010 Cornaredo (Mi), Italy; marco.soldo@st.com

**Keywords:** optical low-coherence reflectometry, optical measurements, thin-film piezo-electric actuator, non-destructive testing

## Abstract

In this work, we have applied optical low-coherence reflectometry (OLCR), implemented with infra-red light propagating in fiberoptic paths, to perform static and dynamic analyses on piezo-actuated glass micro-membranes. The actuator was fabricated by means of thin-film piezoelectric MEMS technology and was employed for modifying the micro-membrane curvature, in view of its application in micro-optic devices, such as variable focus micro-lenses. We are here showing that OLCR incorporating a near-infrared superluminescent light emitting diode as the read-out source is suitable for measuring various parameters such as the micro-membrane optical path-length, the membrane displacement as a function of the applied voltage (yielding the piezo-actuator hysteresis) as well as the resonance curve of the fundamental vibration mode. The use of an optical source with short coherence-time allows performing interferometric measurements without spurious resonance effects due to multiple parallel interfaces of highly planar slabs, furthermore selecting the plane/layer to be monitored. We demonstrate that the same compact and flexible setup can be successfully employed to perform spot optical measurements for static and dynamic characterization of piezo-MEMS in real time.

## 1. Introduction

In the past years, membranes or diaphragms actuated by thin piezo-electric films have been incorporated in various examples of MEMS devices, such as micro-pumps [1], micro-valves [2], micro-mirrors [3], tunable gratings [4], switches [5,6], and micro-optics [7,8,9,10,11]. Thanks to advancements in MEMS technology, piezo-thin-film fabrication processes are now providing devices with performances and reliability that match the characteristics of traditional, though more expensive, bulk piezo-elements.

Optical characterization and testing of MEMS can currently be performed by using standard instrumentation based on different techniques, such as Doppler velocimetry, scanning white light profilometry, phase shift interferometry, and vertical scanning interferometry, or their combinations [12,13]. However, commercially available instruments have a significantly high cost, and most of them can hardly result in a compact implementation. Several instruments are based on sophisticated analyses of detected images: estimation of static and dynamic behaviors usually require different, convoluted schemes, supported by sampling techniques and post-processing. Characterization methods of MEMS devices based on spot optical measurements are able to give quite valuable information, even on single events, in real time with much easier procedures [14,15,16,17,18]. In view of the breakthrough of thin-film technology in MEMS production lines, there is an increasing interest in the development of instrumental configurations with compact setups for device testing, that are able to provide data in real-time on different features of the fabricated micro-devices, possibly with high spatial resolution.

In this work, we report the results of static and dynamic analyses performed on piezo-actuated micro-structures by means of optical low-coherence reflectometry (OLCR), implemented with a compact fiberoptic setup and near infrared (NIR) radiation. The piezo-electric thin-film actuator was fabricated on top of a glass micro-membrane, about 20 μm thick, using the Thin-Film-Process (TFP) MEMS technology developed by STMicroelectronics [19,20,21]. By applying DC or AC voltage, the piezo-electric actuator induced a stress on the edges of the micro-membrane that was thus forced to bend and to assume a curved shape. In OLCR, a low-coherence optical source feeds an optical interferometer in the Michelson scheme, that is external to the source. The input radiation is split and is redirected partially along a measuring arm, towards the device under test, and partially along the reference arm, towards a mirror. Recombination on a photodetector of the back reflected radiation from both arms generates an intensity modulated signal that carries the information on the optical path-length difference between measuring and reference arm. Groups of fringes are developed only when this difference is shorter than the coherence length of the read-out source. The limited time coherence of the readout source, considered a drawback in laser interferometers, becomes very useful when testing multilayer planar structures incorporating multiple parallel interfaces.

We are here demonstrating that spot optical measurements carried out with optical low-coherence reflectometry provide real-time data on the optical path-length of the glass layer, by translating with constant velocity the mirror at the end of the reference arm. Moreover, by fixing the position of the reference mirror to ensure optical path-length matching on both arms, it is possible to quantify the micro-membrane displacement versus the driving voltage applied to the piezo-actuator or the resonance frequency of the MEMS device, simply exploiting different excitation signals and signal processing schemes. In this paper, using an optical source with limited coherence-time, such as a superluminescent light emitting diode with central emission wavelength of approximately 1.3 µm, allows to select the plane/layer to be monitored and to perform high-resolution interferometric measurements of displacement, typical of a coherent detection, without spurious resonance effects that would be generated by the close, parallel interfaces of highly planar micro-slabs.

## 2. Measuring Setup and Piezo-Actuated Glass Micro-Membrane

**Figure 1:**
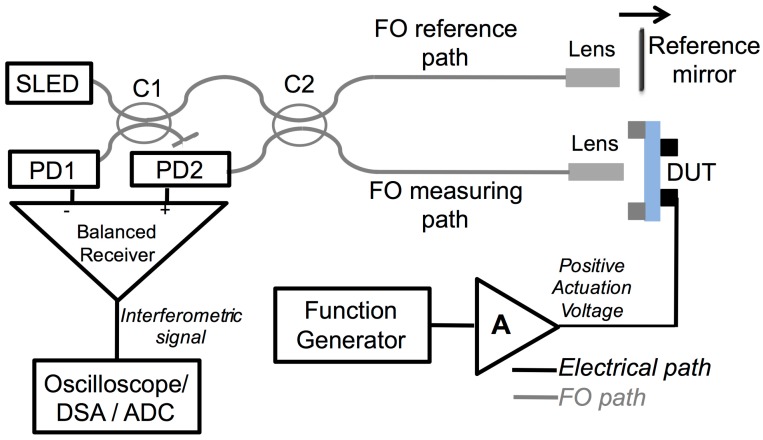
Instrumental and optical configuration of IR OLCR applied for characterizing piezo-actuated micro-membranes (MEMS Under Test). SLED: Superluminescent Light Emitting Diode; PD: Photodiode; C1, C2: 2 × 2, 50:50 fiberoptic coupler: FO: fiberoptic; DSA: Digital Signal Analyzer; ADC: Analog to digital conversion board; A: amplifier.

The instrumental and optical configuration of the applied NIR OLCR, previously described and applied only for static analyses in [22,23], is reported in Figure 1. It consists in a Michelson interferometric scheme with balanced receiver for signal detection. By cascading two FO splitters (C1 and C2 in Figure 1), broadband radiation is partly coupled along the “measuring arm” toward the MEMS under test and along the “reference arm” to a translating reference-mirror. Impinging light is projected on the device under test (and back reflected light is then collected) by means of pigtail style focusers, based on 9/125 core-cladding diameter single-mode fibers, incorporating a f = 3.9 mm aspheric lens, with 40 dB return loss, by OzOptics, USA. Out-of-phase signals, generated by radiation back-reflected from the mirror interfering with radiations back-reflected from the MEMS, are then detected by two InGaAs FGA01FC photodiodes by Thorlabs, USA (PD1 and PD2 in Figure 1). For the application described in this work, broadband radiation is provided by a fiber-coupled Superluminescent Light Emitting Diode (SLED, Covega Thorlabs SLD1021, USA) with Gaussian emission spectrum centered at λ ≈ 1308 nm and full width at half maximum (FWHM) bandwidth ∆λ ≈ 52 nm. The coherence length L_c_ of the SLED is calculated with the relation L_c_ = 0.66 × λ^2^/∆λ, yielding L_c_ ≈ 22 µm. Spatial coherence is ensured by propagation of the fundamental mode in the optical fibers. The output voltage of the balanced receiver can be acquired and processed in different ways, depending on the analysis that is performed and the parameter that is investigated. The MEMS under test consists in a 20-µm-thick glass micro-membrane (1.5-mm diameter) that is anchored all around the borders to a silicon support. A 2-µm-thick piezo-electric (PZT) ring provided with top and bottom electrodes is fabricated on the surface of the membrane. This piezo-actuated glass membrane is also used as structural element of the commercially available poLight TLens^®^ [10,11]. By applying a voltage, the piezo-electric actuator forces out-of-plane displacements of the thin glass membrane; a positive voltage between top and bottom electrodes induces upward bending of the membrane. Measurements were performed in the center of the micro-membrane identified with the help of the red radiation emitted by a HeNe laser coupled into the fiberoptic path.

## 3. Results

### 3.1. Micro-Membrane Optical Path-Length

**Figure 2:**
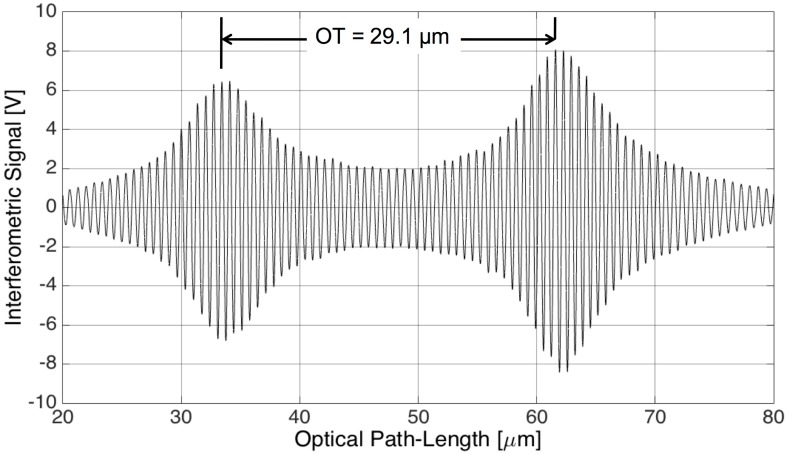
Interferometric signal as a function of optical path-length variation of the reference arm.

With OLCR, the optical path-length or optical thickness (*OT*) of the glass micro-membrane was detected by shining the readout beam in direction orthogonal to the plane of the membrane [22,23]. The interferometric signal for extracting the membrane *OT* was generated by translating the reference mirror (by means of a computer-controlled, motorized translation stage) across the positions where the optical path-length of the reference arm matched the optical path along the measuring arm up to both air-glass interfaces. The interferometric signal was then acquired in the time-domain (*t*) through an analog to digital (ADC) conversion board connected to a personal computer and analyzed in the optical path-length domain. Assuming a constant mirror speed, at least during the acquisition time, the optical path-length was obtained multiplying the acquisition time (*t*) by the traveling speed (*v* = 10 μm/s) of the reference mirror. The typical interferometric signal detected on the glass micro-membrane is reported in Figure 2 as a function of the optical path-length variation on the reference arm, generated by translating the reference mirror. It consists in an amplitude-modulated sinusoidal signal that corresponds to a sequence of fringes, each one due to a target displacement equal to λ/2, where λ is the central wavelength of the SLED. The highest amplitude of the fringe envelope is achieved when the optical path-length on the reference arm is equal to the optical path-length on the measuring arm relative to the air-glass and glass-air interfaces crossed by the readout beam. As the SLED has a Gaussian emission spectrum, also the amplitude of the interferometric fringes close to the interface positions is modulated by a Gaussian envelope. The full width at half maximum of the Gaussian envelopes is ≈11 µm, thus approximately equal to L_c_/2 as expected. The optical thickness *OT* of the glass layer is the optical distance between the interfaces that can be calculated as the distance between the peaks of the fringe envelope. The coherence length of the SLED was sufficiently short for well separating the back-reflected signal relative to each interface allowing us to detect *OT* ≈ 29.1 µm by counting 44.5 fringes. As the optical thickness of the glass layer is proportional to the geometrical thickness *d*, *OT* = *d* × *n_g_*, where *n_g_* is the group refractive index of the material at the center wavelength of the employed broadband source, by assuming *d* ≈ 20 µm, as design parameter, we obtained *n_g_* = 1.46 in agreement with values reported in the literature for similar glasses [24]. No changes in the optical thickness of the glass micro-membrane were observed by applying voltage to the piezo-actuator.

### 3.2. Micro-Membrane Displacement Induced by Quasi-Static Piezo-Actuation

We applied NIR OLCR for displacement measurements of the air-glass interface as a function of the voltage applied to the piezo-actuator in quasi-static conditions. As previously mentioned, the SLED coherence length is sufficiently short to allow separate interrogations of the interfaces of the tested device. At the same time, the SLED provides interferometric signals with an excellent signal-to-noise ratio even when the reference and measuring arm have different optical path-lengths up to ±10 µm. The piezo-actuator was driven with an AC voltage consisting in a triangle wave at 45 Hz, from 0 V up to +40 V. The 45 Hz frequency was more than two decades lower than the lowest resonance frequency of the structure ensuring an effective displacement in phase with the driving signal. The reference mirror position was selected and fixed in order to attain the peak of the fringe group relative to the air-glass interface at approximately 25 V.

**Figure 3:**
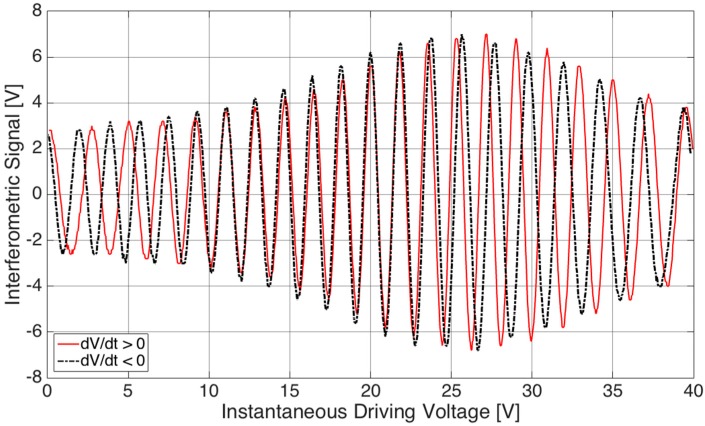
Interferometric signal as a function of the instantaneous driving voltage applied to the piezo-electric actuator. Red trace: results for d*V*/d*t* > 0; black dashed trace: results for d*V*/d*t* < 0.

**Figure 4:**
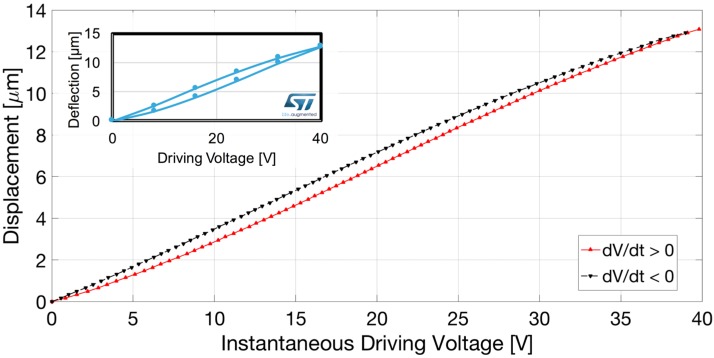
Micro-membrane displacement as a function of the instantaneous driving voltage applied to the piezo-electric actuator, quantified by fringe counting with λ/8 resolution. Red trace: results for d*V*/d*t* > 0; black dashed trace: results for d*V*/d*t* < 0. Inset: Results provided by finite element numerical simulations carried out with COMSOL 4.3.

We acquired both the interferometric signal (output of the balanced receiver) and triangle driving wave in the time domain with a digital oscilloscope. In Figure 3 the interferometric signal is plotted as a function of the instantaneous driving voltage. The red trace refers to the interferometric signal collected for increasing voltage (thus, in the half-period of the triangle wave exhibiting positive slope, d*V*/d*t* > 0) whereas the black dashed trace is for decreasing voltage (thus, in the half-period of the triangle wave exhibiting negative slope, d*V*/d*t* < 0). Hysteresis in the position assumed by the micro-membrane for increasing and decreasing voltages, as expected for piezo-electric actuation [25,26,27], is highlighted by this plot as the fringes for d*V*/d*t* > 0 are not superposed to the fringes detected for d*V*/d*t* < 0. The displacement was finally reconstructed as a function of the instantaneous value of the piezo-actuator driving voltage by fringe counting with a λ/8 = 165 nm resolution, that is by detecting the voltage corresponding to all the maxima, minima and zero crossings of the interferometric signal. The micro-membrane displacement reconstructed within a period of the triangle wave as a function of the instantaneous voltage is shown in Figure 4. The red trace refers to the results obtained for d*V*/d*t* > 0 whereas the black dashed trace is relative to the results attained for d*V*/d*t* < 0. The detected displacement and hysteretic cycle are in agreement with the results provided by finite element modelling and numerical simulations carried out with COMSOL 4.3 Multiphysics 3D Model, shown in the inset of Figure 4. As the diameter of the readout beam in the infrared is approximately 50 µm, the spatial resolution is very high: spot optical measurements performed in a few positions could provide data on the device deformation.

### 3.3. Resonance Curve of the Fundamental Out-of-Plane Vibration Mode

**Figure 5:**
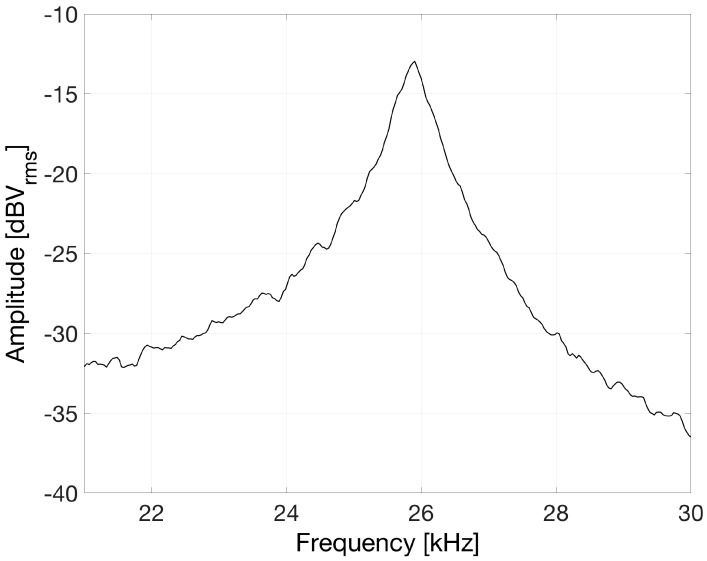
Frequency response of the fundamental out-of-plane vibration mode of the micro-membrane obtained by driving the piezo-actuator with electrical white noise.

We finally applied OLCR for characterizing also the dynamic response of the piezo-actuated micro-structure and obtaining the resonance curve of the fundamental out-of-plane vibration mode. Using the SLED as readout source, OLCR provided interferometric signals with an excellent signal-to-noise ratio also when operated in quadrature (that is with a π/2 phase difference between the reference and the measuring fields). In these operating conditions, the interferometric signal is usually assumed proportional to the vibration amplitude of the device under test, at least for displacements smaller than *λ*/2. For resonance curve detection, the piezo-actuator was driven by white electrical noise, at least in the band of interest, superposed to a positive DC component. The output signal of the balanced receiver was fed to a Dynamic Signal Analyzer that provided the frequency response by performing the Fast Fourier Transform of the signal. The result reported in Figure 5 shows a well-defined resonance peak at 25.9 kHz, in agreement with the results of numerical simulations and of measurements performed with a Polytec MSA-500 Micro System Analyzer, available in STMicroelectronics laboratories.

## 4. Conclusions

We demonstrated the functionality of NIR OLCR, implemented with a compact, all-fiber setup, for monitoring the out-of-plane actuation of a glass micro-membrane, applied by means of a piezo-electric film. The same basic optical setup was successfully employed for static and dynamic characterization. In addition to static measurements of optical thickness, also reported in previous papers on different devices [22,23,28], we investigated out-of-plane large displacements of the micro-membrane at low frequency to identify hysteretic effects induced by positive and negative slopes of the driving voltage. Given the applied instantaneous voltage, it is possible to immediately quantify the hysteresis, thanks to the amplitude modulation of the sinusoidal interferometric signal induced by the Gaussian broad emission spectrum of the SLED. Thus, the use of a low-coherent optical source such as the SLED enables to solve the 2π ambiguity typical of laser interferometer for displacement measurements. We also tested the dynamic behavior of the structure by detecting the frequency of the fundamental vibration mode that was found in the kHz range. A peculiar property of OLCR consists in the possibility to select the monitored layer, an interesting feature for testing multilayer devices. Moreover, the use of an optical source with low time coherence avoids any resonance effects due to the parallel interfaces of highly planar slabs that could eventually disturb laser interferometers. With regards to limitations, OLCR does not provide the geometrical distance between interfaces but only the optical path-length in the NIR. As implemented in our setup, it does not provide the absolute distance of the first crossed interface from the output lens; however, this issue could be solved by adding a reference plane for static measurements, for example by placing a thin glass slide on the back of the MEMS device. Although it is possible to scan the surface for mapping the membrane displacement in several positions, thanks to the small spot size of the readout beam, when the curvature of the membrane is significantly increased (the radius of curvature is decreased), coupling losses tend to increase when moving from the center towards the edges of the membrane.

In conclusion, the implemented fiberoptic NIR OLCR setup is a powerful and highly versatile diagnostic tool for in-depth, non-destructive testing of multilayer MEMS in real-time, as its performances and applications can be tailored by proper selection of the optical source as well as of the method for signal processing. Measurements of mechanical displacements taken on the boundary of the piezoelectric structure and performed with our setup may also become useful responses for identifying material interfaces, for example due to inclusions, by inverse problem solving using the level set algorithm [29].
